# The Altered Hepatic Tubulin Code in Alcoholic Liver Disease

**DOI:** 10.3390/biom5032140

**Published:** 2015-09-18

**Authors:** Jennifer L. Groebner, Pamela L. Tuma

**Affiliations:** Department of Biology, The Catholic University of America, Washington, DC 20064, USA; E-Mail: 41groebner@cardinalmail.cua.edu

**Keywords:** microtubules, tubulin, acetylation, post-translational modifications, ethanol, hepatocytes, liver

## Abstract

The molecular mechanisms that lead to the progression of alcoholic liver disease have been actively examined for decades. Because the hepatic microtubule cytoskeleton supports innumerable cellular processes, it has been the focus of many such mechanistic studies. It has long been appreciated that α-tubulin is a major target for modification by highly reactive ethanol metabolites and reactive oxygen species. It is also now apparent that alcohol exposure induces post-translational modifications that are part of the natural repertoire, mainly acetylation. In this review, the modifications of the “tubulin code” are described as well as those adducts by ethanol metabolites. The potential cellular consequences of microtubule modification are described with a focus on alcohol-induced defects in protein trafficking and enhanced steatosis. Possible mechanisms that can explain hepatic dysfunction are described and how this relates to the onset of liver injury is discussed. Finally, we propose that agents that alter the cellular acetylation state may represent a novel therapeutic strategy for treating liver disease.

## 1. Introduction

In 2012, 3.3 million deaths worldwide were attributed to alcohol consumption which translates to 5.9% of all global deaths (7.6% for men and 4.0% for women) [1]. Approximately 90% of these deaths are due to alcohol-attributable cardiovascular disease, cancer and gastrointestinal disorders (mainly liver cirrhosis) [1]. Despite considerable research efforts aimed at understanding the clinical presentation and progression of alcoholic liver disease, the specific mechanisms leading to alcohol-induced tissue damage remain elusive. Defining these cellular mechanisms is critical for the development of more effective treatments for patients suffering from alcoholic liver disease. For well over three decades, the hepatic microtubule cytoskeleton has been the focus of mechanistic studies to explain observed alcohol-induced defects in hepatocyte processes. One of the original observations was that *in vitro*, α-tubulin is preferentially acetaldehyde adducted on a highly reactive lysine which leads to drastically impaired microtubule polymerization [2,3,4,5,6,7] (see below). This was confirmed in isolated hepatocytes from alcohol-fed rats where microtubule regrowth after treatment with nocodazole (a reversible, microtubule poison) was found to be significantly impaired [8]. Understanding how impaired polymerization relates to progression of liver injury remains the subject of continued studies, but because microtubules are central to a myriad of cellular processes ranging from protein trafficking to mitosis, other features of the microtubule network are also actively being examined.

Microtubules are characterized by a host of post-translational modifications (PTMs) that was dubbed the “tubulin code” in 2007 [9]. For comprehensive, recent discussions on the current understanding of the tubulin code, we refer readers to the following excellent reviews [9,10,11,12]. In this review, we will briefly describe the tubulin code, but also emphasize how it relates to hepatocyte function in health and in the alcoholic liver. We propose that ethanol exposure alters the tubulin code leading to impaired microtubule-based processes ultimately leading to liver injury. We also describe how such studies may lead to novel therapeutic approaches to treating alcoholic liver disease.

## 2. The Tubulin Code

### 2.1. Modifications of the Tubulin Code

Microtubules are made of repeating units of α- and β-tubulin heterodimers that form protofilaments, which in turn assemble into hollow tubes consisting of 13 protofilaments arranged in parallel. Microtubules exist as both dynamic and stable polymers [13]. Unlike dynamic microtubules, the stable population is characterized by a longer half-life (1 h *vs*. 10 min), resistance to microtubule poisons (e.g., cold and depolymerizing agents like nocodazole) and by specific PTMs (Table 1). The majority of the modifications are present on the extreme, extending C-terminal domains of the subunits and include the enzymatic removal of the carboxy-terminal tyrosine (detyrosination), additional removal of the penultimate glutamate (∆2), mono/polyglutamylation and mono/polyglycylation. Another well-characterized modification closer to the N-terminal domain is acetylation of lysine 40 on α-tubulin [14]. Other less common or less-studied modifications include polyamination, *O*-linked glycosylation, palmitoylation, phosphorylation, sumoylation, ubiquitination and succination (Table 1). Among these minor modifications, glycosylation, phosphorylation, sumoylation and ubiquitination have been shown to be restricted to the soluble dimer and in many cases prevent microtubule polymerization.

In general, the modifications of polymeric tubulin are present on the stable microtubules, and in practice are used to “mark” the stable population. However, despite the many studies devoted to examine the effect of the various modifications on polymer stability, there is no definitive evidence that the PTMs confer microtubule stability, subunit turnover or alter the intrinsic properties of the polymer [9,10,11,12]. Instead, the current hypothesis is that the various PTMs alter overall microtubule structures that ultimately leads to changes in interactions with motors, microtubule associated proteins (MAPs) and other cellular structures and effectors. Thus, like for the protruding N-terminal PTMs of the histone code that promote alterations in chromatin structure and function, the tubulin PTMs represent a “code” that is interpreted by other proteins leading to changes in microtubule-based processes [9]. However, unlike for the histone code, the tubulin PTMs are likely not inherited, but rather are reestablished in each new cell or emerging microtubular structure (e.g., primary cilium, centriole) [9].

### 2.2. Acetylation Is the Primary Modification in Hepatocytes and It Is Enhanced upon Ethanol Exposure

The majority of the microtubule modifications are present on specialized microtubules such as centriolar tubulin or the axonemal microtubules present in cilia and flagella (Table 1). Axonal and dendritic microtubules are also highly modified and many PTMs have only been observed or are highly enriched in neurons. For example, only axonal microtubules have been shown to be polyaminated [15]. In contrast, cytoplasmic microtubules are associated with far fewer modifications restricted mainly to acetylation and detyrosination, and in some cases monoglutamylation. Unlike most polarized epithelial cells, hepatocytes lack primary cilia, such that only centriolar and cytoplasmic microtubule subpopulations are present [16]. Both detyrosinated and glutamylated microtubules are detected in centriolar structures in hepatocytes as for other cell types [17]. Although these modifications have also been shown to predominate on cytoplasmic microtubules in polarized epithelial cells upon differentiation [18,19], they have not been detected on polymeric tubulin in hepatocytes [17]. To date, acetylation is the only PTM detected on cytoplasmic microtubules in hepatocytes [17,20], and it is this modification that is enhanced in ethanol-exposed cells [20].

Over 10 years ago, we determined that alcohol exposure leads to increased microtubule acetylation on lysine 40 of α-tubulin to approximately 3-fold over that of control in polarized WIF-B cells [20]. Importantly, WIF-B cells are fully differentiated hepatic cells that retain the ability to metabolize alcohol with endogenously expressed enzymes [21]. As for other *in vitro* and cell culture systems, microtubule polymerization was also impaired in ethanol-treated WIF-B cells [20]. However, once the polymer was established, we found it was relatively rapidly and extensively acetylated [20]. We further determined that increased acetylation correlates with increased microtubule stability and confirmed these findings in VL-17A cells, liver slices and in livers from ethanol-fed rats indicating the findings have physiologic importance [20,22].

As interest in the acetylome has increased, many comprehensive proteomic studies have been performed to define the acetylated proteins present in different mammalian cell types (including mouse liver). In nearly all of these mass-spectroscopy-based studies, multiple lysines in peptides derived along the length of both α and β-tubulin have been identified [23,24,25]. Similarly, multiple acetylated lysines in the motor proteins and their accessory factors (kinesin and the various dynein and dynactin subunits) have also been identified [23,24,25,26]. To date, the presence of the modified residues has not been confirmed *in vivo* nor has their functional significance been determined. Nonetheless, it is compelling to speculate that at least a subset of these residues are hyperacetylated in the ethanol-exposed hepatocyte and contribute to altered intracellular motility.

**Table 1:** The post-translational modifications of the tubulin code.

Major PTM	α/β	Site	Distribution	References	Hepatocytes	References
Acetylation	α	Lys40	Centrioles, midbodies, mitotic spindles, neurons, cilia, flagella, cytoplasmic microtubules	[27,28,29,30,31,32,33]	Cytoplasmic microtubules	[17,20]
	β	Lys252	Soluble dimer	[34]		
Detyrosination	α	C-terminal Tyr removal	Centrioles, midbodies, mitotic spindles, neurons, cilia, flagella, cytoplasmic microtubules	[28,29,35,36,37,38,39,40,41,42]	Centrioles (?)	[17]
Deglutamylation (Δ2-tubulin)	α	C-terminal Glu removal from detryrosinated CTTs	Centrioles, neurons, cilia, flagella	[43,44,45,46]		
Mono/poly-Glutamylation	α/β	Glu(s) addition to Glu in CTTs	Centrioles, midbodies, mitotic spindles, neurons, cilia, flagella, cytoplasmic microtubules (mono only)	[47,48,49,50,51,52,53,54,55,56,57]	Centrioles (?)	[17]
Mono/poly-Glycylation	α/β	Gly(s) addition to Glu in CTTs	Cilia, flagella	[35,58,59,60]		
**Minor PTM**		**Comments**			**References**	
Polyamination		Found only in neurons; Gln15 in β-tubulin and other unidentified α- and β-tubulin sites			[15]	
*O*-linked Glycosylation		Examined only in neurons, B lymphocytes, HeLa cells, L6 myotubes and MN9D neuronal cells; various unidentified α- and β-tubulin sites			[61,62,63,64]	
Palmitoylation		Examined only in neurons (Cys376 in α-tubulin) and in yeast (Cys377 in α-tubulin)			[65,66,67]	
Phosphorylation		Examined only in neuroblastoma cells, rat brain and COS-7 cells; various unidentified α- and β-tubulin sites and Ser172 in soluble β-tubulin			[68,69,70,71,72]	
Sumoylation		Examined only in yeast and HEK293 cells (overexpressing SUMO); multiple unidentified α-tubulin Lys			[73,74]	
Ubiquitination		Examined only in neurons, cilia, flagella, and HEK293 cells (overexpressing Parkin); multiple unidentified α-tubulin Lys			[75,76,77]	
Succination		Examined in adipocytes, C2C12 myotubes grown in high glucose and adipose tissue of *db/db* diabetic mice; Cys347 and 376 in α-tubulin, Cys12 and 303 in β-tubulin			[78]	

The major and minor PTMs of the tubulin subunits are listed. The specific sites on α- or β-tubulin that are modified (if identified) are also indicated. For the major PTMs, the microtubule subpopulations that contain the specific modifications are listed, and what is known about the modifications in hepatocytes is indicated. For the minor PTMs, brief comments are provided about what is known about these less well-studied modifications. References, references cited; CTTs; C-terminal tails.

### 2.3. Other Ethanol-Induced Modifications of Microtubules

**Figure 1:**
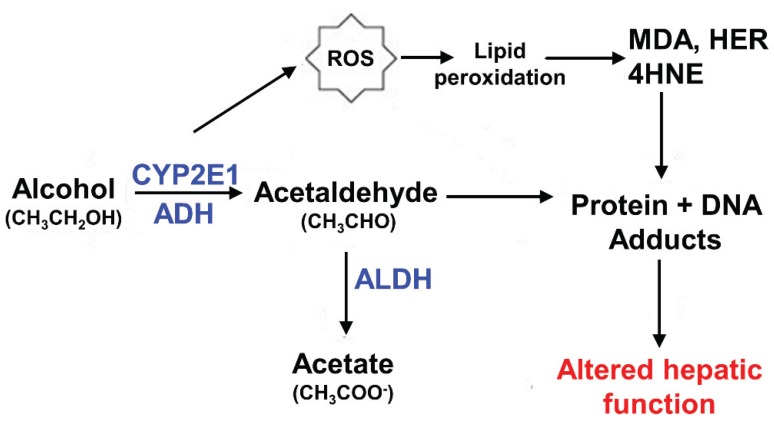
Hepatic ethanol metabolism. Alcohol metabolism by alcohol dehydrogenase (ADH) and cytochrome P450 2E1 (CYP2E1) results in the formation of the reactive intermediate, acetaldehyde. CYP2E1-mediated metabolism also produces reactive oxygen species (ROS) and hydroxyethyl radicals (HER) that lead to enhanced oxidative stress and lipid peroxidation. The modified lipids are further metabolized to other highly reactive intermediates including malondialdehyde (MDA) and 4-hydroxy-2-nonenal (4-HNE). All of these highly reactive metabolites form adducts with proteins, lipids and DNA that impair proper hepatic function.

In addition to the modifications of the natural repertoire, hepatic tubulin subunits are also modified by the many reactive metabolites produced by ethanol metabolism. In the hepatocyte, ethanol is converted to acetaldehyde by alcohol dehydrogenase (ADH) (Figure 1). This highly reactive intermediate is further metabolized in mitochondria to acetate by acetaldehyde dehydrogenase (ALDH). Alcohol is also metabolized by cytochrome P_450_ 2E1 (CYP2E1) leading to the formation of acetaldehyde, but also to the formation of oxygen and hydroxyethyl radicals that in turn promote the formation of other reactive intermediates (for review see [79]).

Over three decades ago, it was found that α-tubulin was a major target for adduction by acetaldehyde [7]. *In vitro*, this subunit purified from either bovine brain [2,80] or rat liver [3] was found to be preferentially modified on a highly reactive lysine (maybe lysine 394) [4,5] in a time and concentration dependent manner [2,3]. This reactive lysine is more accessible to adduction on soluble tubulin, thus unlike for acetylation, polymeric α-tubulin is less susceptible to modification by acetaldehyde [2,3]. *In vitro* polymerization assays using low acetaldehyde:tubulin dimer levels further revealed that adduction drastically impaired microtubule formation [80]. This impairment occurred at substoichiometric amounts of acetaldehyde (0.2 moL acetaldehyde/moL tubulin) [81] suggesting that low levels of adduction can have far reaching effects on microtubule function. Furthermore, acetaldehyde robustly binds purified hepatic MAPs and motors, 1.5-fold more than tubulin [2] which also likely contributes to altered cellular function.

More recently, various proteomic studies have also revealed that tubulin can be adducted by products of oxidative stress in the alcoholic liver or other pathological states. For example, both α and β-tubulin are carbonylated in livers from ethanol-fed mice [82] or s-nitrosylated in brains from mice with experimental autoimmune encephalomyelitis [83] or Alzheimer’s disease [84]. *In vitro*, tubulin is readily adducted on lysines by the lipid peroxidation products, 4-hydroxynonenal (4-HNE) and 4-oxononenal (4-ONE) [85]. Furthermore, amino acids in tubulin have been shown to be readily non-enzymatically modified to atypical isoaspartyl residues, a sign of protein damage [86]. Based on these results, we predict that multiple tubulin residues are adducted by these and other metabolites such that the tubulin code is even more complicated in alcohol-exposed hepatocytes leading to even a greater potential for dysfunction in microtubule-based processes. Further complicating matters is the likelihood of similar adducts on the microtubule motors and MAPS. For example, subunits of the dynactin complex are highly oxidized in livers from ethanol-fed mice [87].

An important open question is the stoichiometry of the modifications by ethanol metabolites and those of the natural repertoire *in vivo*. At present, little is known about the steady state stoichiometries of the individual modifications or how they vary with time, level of differentiation or in the pathological state. Acetylation of lysine 40 is apparently sub-stoichiometric *in vivo* since its levels can be increased 2-3-fold by ethanol exposure. Interestingly, this fold-increase is saturable and does not increase with increased days in ethanol or by increased concentrations of ethanol, yet addition of trichostatin A (a pan deacetlyase inhibitor) leads to saturable microtubule acetylation to much higher levels of 10-12-fold over steady state levels [88]. Thus, strict mechanisms must be in place to maintain steady state modification levels. It will be important to determine the specific stoichiometries of each modification to better understand the cellular phenotypes and pathologies associated with modified microtubules.

Another additional and important feature of the tubulin code in ethanol-exposed liver is that enhanced microtubule acetylation requires ethanol metabolism. Microtubule acetylation displays a saturable, dose and time-dependence on ethanol addition [20]. Studies with 4-methylpyrazole (4-MP), an ADH inhibitor (that leads to decreased cellular acetaldehyde levels), and cyanamide, an ADLH inhibitor (that leads to increased cellular acetaldehyde levels) further suggest that microtubule acetylation is mediated by acetaldehyde [20]. How acetaldehyde promotes microtubule acetylation is not completely understood, but a possible answer might come from our studies on the hepatic microtubule deacetylase (see below). Also, it is not known to what extent acetaldehyde adduction and acetylation “compete” for lysine residues. From our recent proteomics analysis of tubulin isolated from control and ethanol-treated cells, we determined that acetylation and acetaldehyde adduction can occur on the same lysine residues (e.g., on lysine 40) (manuscript in preparation). Furthermore, peptides from the same alcohol-exposed tubulins have either no modifications at lysine 40, were acetylated or were acetaldehyde adducted (manuscript in preparation). So, clearly the modifications can “compete” but who wins is not clear and their relative stabilities as microtubules undergo dynamic instability and repolymerization are also not known. Finally, addition of CYP2E1 inhibitors or anti-oxidants does not alter microtubule acetylation in ethanol-exposed hepatic cells (our unpublished results, [89]) indicating the modification is not dependent on metabolites of oxidative stress.

### 2.4. A Possible Mechanisms for Ethanol-Induced Microtubule Acetylation

Lysine acetylation is regulated by the coordinated activities of acetyltransferases and deacetylases. α-tubulin acetyltransferase-1 (αTAT-1) has recently been identified as the specific microtubule acetyltransferase [90,91]. Both histone deacetylase 6 (HDAC6) and sirtuin T2 (SirT2) have been shown to deacetylate tubulin in a variety of cell types [92,93,94,95], but immunoblotting, immunofluorescence microscopy, and assays using the SirT2 inhibitor, nicotinamide (NADH is a SirT2 required cofactor), revealed that polarized WIF-B cells (and hepatocytes?) do not express SirT2 [96]. In contrast, HDAC6 is highly expressed in WIF-B cells and likely accounts for microtubule deacetylation in hepatic cells [96]. Changes in the expression of either αTAT or HDAC6 or in their catalytic activities have been correlated with changes in tubulin acetylation. Thus, a simple explanation for ethanol-induced protein acetylation may be altered enzyme levels or activities. Although αTAT-1 has not yet been examined in ethanol-treated cells, we determined that HDAC6 levels were moderately decreased by ~25% in ethanol-treated WIF-B cells and that its catalytic activity was not changed [96] such that the simple predictions do not explain the impaired microtubule deacetylation.

However, direct binding assays revealed that HDAC6 binding to microtubules, was impaired by ~70% in ethanol-treated cells correlating with decreased acetylation. However, HDAC6 from ethanol-treated cells readily deacetylated added exogenous porcine brain tubulin suggesting no changes in enzyme-substrate interactions. This suggests that tubulin from ethanol-treated cells was modified thereby preventing HDAC6 binding and subsequent deacetylation [96]. Like enhanced acetylation, impaired HDAC6 binding to microtubules requires ethanol metabolism, and from studies using 4-MP, is likely mediated by acetaldehyde [20,96]. Thus one provocative possibility is that tubulin acetaldehyde adducts impede HDAC6 binding in ethanol-treated cells. It is not yet known whether adduction directly prevents HDAC6 binding to a specific motif or whether it results in conformational changes that abolish the substrate binding site. Nonetheless, these results can explain why CYP2E1-induced ethanol metabolism is not associated with enhanced microtubule acetylation—the ROS-associated adducts simply do not interfere with HDAC6 binding to acetylated sites. Future studies are needed to test this possibility directly and to examine similar αTAT-1 properties in ethanol treated cells.

## 3. Consequences of Altered Microtubule Modifications on Cellular Function

### 3.1. Impaired Protein Trafficking

**Figure 2:**
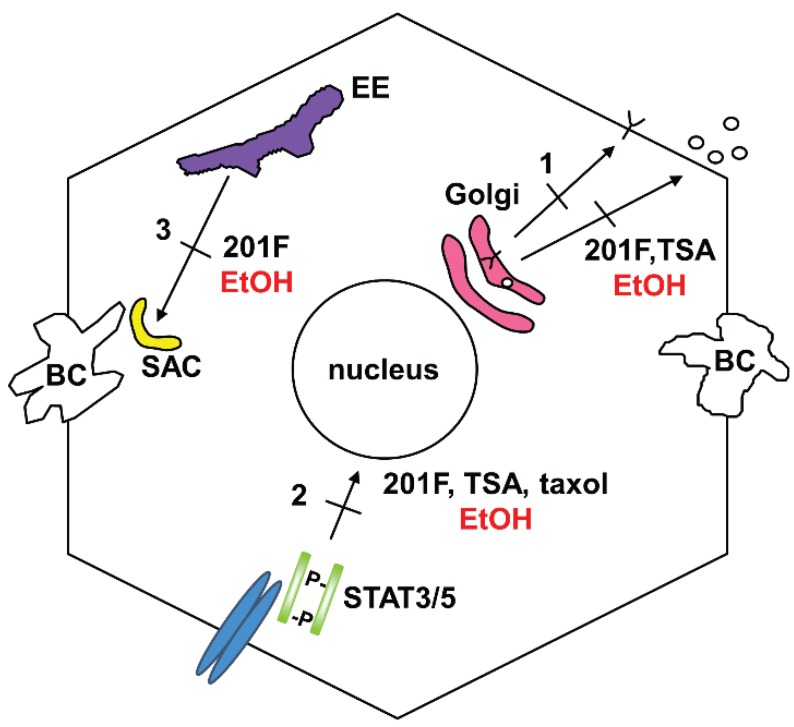
Alcohol-induced defects in hepatic trafficking that are microtubule-dependent. Secretion and delivery of newly synthesized basolateral proteins are impaired in ethanol-exposed hepatocytes (**1**). Nuclear translocation of selected transcription factors (**2**) and basolateral to canalicular transcytosis (**3**) are also impaired. All steps are also impaired by agents that induce microtubule acetylation in the absence of ethanol. BC, bile canaliculus; SAC, sub-apical compartment, EE, early endosome; TSA, trichostatin A.

To date, a definitive function for microtubule acetylation has not been identified. However, work from almost 20 years ago suggested that different microtubule populations (and/or their modifications) support specific protein trafficking steps [97]. Of particular interest are more recent studies performed in WIF-B cells that used a novel microtubule depolymerizing drug, 201-F [17]. This drug specifically depolymerizes dynamic microtubules leaving only stable, acetylated polymers behind. So far, 201-F-treatment has been shown to impair three microtubule-dependent protein trafficking pathways: basolateral to canalicular transcytosis, basolateral secretion from the TGN and the nuclear translocation of STAT5B [17,98] (Figure 2). Remarkably, all three of these trafficking pathways are also known to be impaired in ethanol-treated hepatic cells (reviewed in [99]) (Figure 2). We have further correlated the ethanol-induced defects in secretion and nuclear translocation to increased microtubule acetylation and stability using two other pharmacological agents: trichostatin A (that induces global protein acetylation) and taxol (that specifically induces microtubule acetylation) [22,32]. In cells treated with these agents to induce similar levels of tubulin acetylation as in ethanol-treated cells (2-3-fold enhancement), secretion and nuclear translocation were impaired to similar extents as that seen in cells treated with ethanol. Together these results strongly suggest that increased microtubule acetylation and stability explain, in part, the alcohol-induced defects in protein trafficking.

### 3.2. A Possible Relationship between Acetylated Microtubules and Alcohol-Induced Steatosis

**Figure 3:**
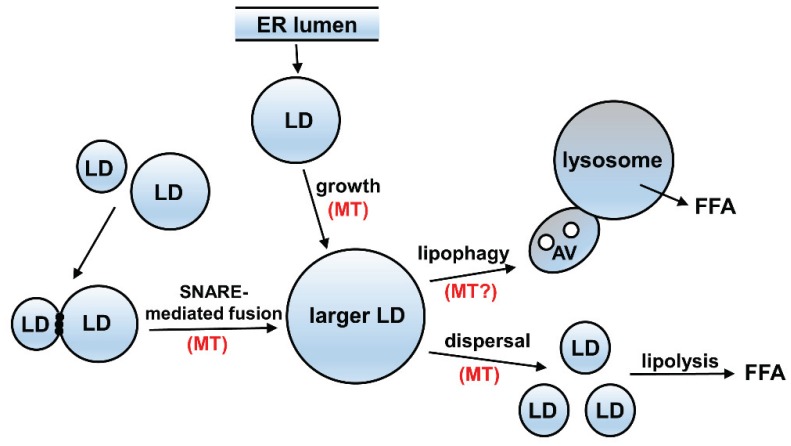
Most steps of the lipid droplet lifecycle are microtubule dependent. Lipid droplets (LDs) released from the ER become larger by direct lipid delivery (growth) or by homotypic fusion. LDs are consumed via lipophagy where autophagic vacuoles (AV) containing engulfed LD fragments ultimately fuse with lysosomes and the hydrolyzed free fatty acids (FFA) are released. Alternatively, LD are dispersed before lipolysis. Not only do microtubules (MT) support dynein and kinesin based LD motility, they are also required for LD growth and fusion. In adipocytes, acetylated MTs vastly enhance lipogenesis. MTs are also required for lipolysis likely by supporting LD dispersal. The role MTs play in lipophagy is not yet known.

Fatty liver (steatosis) is an early stage of alcoholic liver disease that is characterized by hepatocytes with enlarged cytoplasmic lipid droplets. Although the enlarged droplets are considered hepatotoxic, surprisingly little is known about the cellular mechanisms regulating their formation and degradation. Comparison of over a dozen droplet proteomes has revealed that the core machineries that regulate these processes are highly conserved across cell types, tissues, and organisms ranging from bacteria to humans [100,101]. The emerging model of the lipid droplet life cycle is shown in Figure 3. It is now generally accepted that lipid droplets bud from the ER with a phospholipid monolayer encasing neutral lipids (reviewed in [100,102]). The released droplets enlarge by continued lipid delivery from the ER (growth) or by homotypic, SNARE-mediated fusion [103]. Lipid droplets are consumed via lipophagy where autophagic vacuoles containing engulfed droplet fragments ultimately fuse with lysosomes where lipids are hydrolyzed [104]. Alternatively, the droplets are first dispersed before lipolysis [100,102].

Several recent lines of evidence indicate that microtubules are critical for regulating the lipid droplet life cycle at nearly every step. Because tubulin has been identified in almost all proteomes of purified droplets (including from liver [105]) it is considered a core droplet component [100,101]. Also, droplets are highly motile structures and their bidirectional motility requires intact microtubules and is mediated by the microtubule motor proteins, dynein or kinesin [106,107]. Both droplet growth and fusion require intact polymers with the latter preceded by the dynein-mediated clustering of droplets [108,109,110]. Also, droplet dispersion requires microtubules and is supported by motor proteins [110]. Very recently, acetylated microtubules have been shown to be required for adipogenesis in 3T3-L1 cells [111] and that tubulin acetyltransferase αTAT-1 activity is regulated by AMP-activated kinase (AMPK)-mediated phosphorylation [112]. This is particularly compelling since AMPK is considered a master regulator of lipid homeostasis and that its alcohol-induced inactivation has been linked to hepatic steatosis [113,114]. Thus, we propose that alcohol-induced microtubule acetylation leads to altered lipid droplet dynamics thereby contributing to enhanced steatosis.

## 4. Possible Mechanism of Impaired Microtubule-Mediated Processes

Hepatic cell surface polarity is reflected in the asymmetric organization of the cytoplasmic microtubules. Unlike in non-polarized cells where microtubules emanate from a juxta-nuclear microtubule organizing center, there is accumulating evidence that in polarized cells, microtubules are additionally organized from sites at or near the canalicular membrane [115,116]. The emanating microtubules are oriented with their minus ends at the canalicular surface and their plus ends attached to or near the basolateral membrane. Microtubule-based motors mediate vesicle translocation either toward the canalicular surface (toward the microtubule minus-ends) or toward the basolateral surface (toward the plus-ends). In general, all minus-end directed motility is under the regulation of cytoplasmic dynein [117,118]. Although there are a host of plus-end directed kinesin motors, secretion is under the control of kinesin-1 [117,118]. In general, motors are ATPases that physically translocate cargo along microtubules. However, they require additional factors for cargo selection and ATPase activation. The 11-subunit complex, dynactin, is an obligate binding partner for dynein and is required for minus-end directed cargo delivery [118] whereas kinesin likely has numerous effectors.

Recently, we determined that dynein/dynactin colocalizes with stalled transcytosing vesicles at sites of acetylated microtubules in ethanol-exposed cells and that dynein binds more tightly to the hyperacetylated microtubules [119]. The enhanced punctate colocalization of the trafficking canalicular proteins and dynein/dynactin complexes with acetylated tubulin suggests that the transcyotic vesicles are stalled in ethanol-treated cells at sites of hyper-modified microtubules. This is consistent with live cell imaging studies in isolated hepatocytes from ethanol-treated rats where a significant decrease in vesicle motility was observed [120]. Because no differences in dynein protein expression levels, membrane association or ATPase activity were observed in the ethanol-exposed hepatocytes [120], the authors were unable to account for the impaired motility. These findings suggest that ethanol-induced microtubule acetylation and/or adduction may contribute to the defect. Thus, we propose that enhanced dynein binding to modified microtubules in ethanol-treated cells leads to decreased motor processivity resulting in vesicle stalling and in impaired delivery.

Although there is an expanding list of proteins that are known to be hyperacetylated upon ethanol exposure [121], little is known about the functional consequences of this modification. The added acetyl group likely neutralizes the positive charge on lysine while increasing the overall size and hydrophobicity of the side chain. Such changes may result in protein conformational changes that alter function. However, recent structural studies have indicated that acetylated lysine 40 in α-tubulin resides in the microtubule lumen [122] presenting a paradox. Furthermore, *in vitro* cryo-electron microscopy studies comparing maximally deacetylated to maximally acetylated tubulins revealed no differences in tubulin dimer structure or assembly of taxol-stabilized protofilaments [123]. Nonetheless, differences in binding of microtubule motors to acetylated microtubules have been clearly documented. For example, our studies and studies from others have shown that both dynein and kinesin preferentially bind acetylated microtubules [124,125,126]. At present these disparate results cannot be reconciled, but one possible explanation is that lumenal acetylation leads to highly localized conformational changes that ultimately impact surface conformation that alter motor/MAP binding. Such local changes may be masked by taxol stabilization in the ultrastructural studies. Furthermore, these studies do not take into account the other putative sites identified by proteomics screens that are on the polymer surface thereby properly positioned to interfere with motor/MAP binding. Also paradoxically, enhanced microtubule acetylation has been correlated with enhanced kinesin-based neuronal anterograde transport [125], but *in vitro* studies with purified proteins demonstrated that tubulin acetylation *alone* cannot enhance kinesin velocity or run length [127]. Clearly, additional studies are needed to determine what modification or combination of modifications lead to impaired protein trafficking in ethanol-treated cells.

It is also important to note that it has long been appreciated that mutations in tubulin, dynein and kinesin are associated with a host of ciliopathies, neurodegenerative diseases and other forms of neuronal injury (reviewed in [128,129,130]). The myriad of disease phenotypes results from two major defects. The first is that the mutations in tubulin or the motors result in improper microtubule remodeling needed for cell migration or reorganization that allow for processes such as axonal guidance, cellular development or responses to stress. A subset of such cytoskeletal alterations has been correlated to changes in microtubule post-translational modifications, including acetylation. Of note are studies in endothelial cells where microtubule acetylation may prevent necessary polymer reorganization in response to atherogenic stresses [131]. Second, the mutations are also associated with improper protein trafficking resulting in either protein mistargeting or in intracellular protein accumulation and aggregation. The impaired protein trafficking is thought to be due to intrinsic alterations in motor activity or to impaired motor binding to microtubules and/or cargo. In either case, the accumulation of protein aggregates observed in many neurodegenerative diseases (e.g., Alzheimer’s, Huntington’s, ALS) is considered an early and *causative* event in disease progression [128]. Thus, it is compelling to speculate that the impaired motor function and altered microtubule dynamics in ethanol-treated hepatocytes have similar causal relationships with the onset of alcoholic liver disease. This hypothesis is further supported by the finding that in the *Loa* mouse model, the F580Y mutation in dynein heavy chain prevents motor processivity which in turn leads to late onset neurodegeneration [132]. Thus, similar defects in dynein processivity that we propose is occurring in ethanol-treated hepatic cells may also lead to alcoholic liver disease.

## 5. Clinical Significance of Altered Microtubule Post-Modifications and Potential Therapeutics

Although progression of alcoholic liver disease is clinically well-described proceeding from steatosis to fibrosis and finally to cirrhosis, there are no treatments available for the alcoholic patient to alleviate or reverse the disease state. Thus, we argue it is important to actively investigate other possible mechanisms that contribute to the observed clinical pathologies or may contribute to other, less understood mechanisms of hepatotoxicity that may then be targeted for the development of novel therapeutic strategies. Our studies importantly suggest that modulating cellular acetylation levels is one such unique therapeutic target. To date, the acetylation of numerous proteins is known to be enhanced by ethanol-treatment [121]. Currently, specific naturally-occurring and synthetic deacetylase agonists and acetyltransferase antagonists are well-tolerated in humans and are in clinical trials for treatment of inflammation, metabolic disorders (e.g., type II diabetes), cardiovascular and neurodegenerative diseases [133,134,135,136]. One such agent, resveratrol, has also been shown to attenuate fatty liver and oxidative stress in alcohol-exposed mice [137]. In general, the various pharmacological agents are targeted against nuclear HATs or HDACs and likely act by ultimately altering gene expression. Because alcohol induces the hyperacetylation of a host of mitochondrial and cytosolic proteins [121,138,139], the identification of agonists to the major cytoplasmic (HDAC6) or mitochondrial (SirT3, 4 and 5) deacetylases may alleviate oxidative stress or protein trafficking defects without altering gene expression thereby potentially reducing side effects.
